# Co-expression of *CD21L* and *IL17A* defines a subset of rheumatoid synovia, characterised by large lymphoid aggregates and high inflammation

**DOI:** 10.1371/journal.pone.0202135

**Published:** 2018-08-16

**Authors:** Kelly J. McKelvey, Melanie J. Millier, Terence C. Doyle, Lisa K. Stamp, John Highton, Paul A. Hessian

**Affiliations:** 1 Department of Medicine, Dunedin School of Medicine, University of Otago, Dunedin, New Zealand; 2 Department of Medicine, University of Otago, Christchurch, New Zealand; Universitatsklinikum Freiburg, GERMANY

## Abstract

**Objective:**

To determine whether the expression of *IL17A* and *CD21L* genes in inflamed rheumatoid synovia is associated with the neogenesis of ectopic lymphoid follicle-like structures (ELS), and if this aids the stratification of rheumatoid inflammation and thereby distinguishes patients with rheumatoid arthritis that might be responsive to specific targeted biologic therapies.

**Methods:**

Expression of *IL17A* and *CD21L* genes was assessed by RT-PCR, qRT-PCR and dPCR in synovia from 54 patients with rheumatoid arthritis. A subset of synovia (n = 30) was assessed by immunohistology for the presence of CD20^+^ B-lymphocytes and size of CD20^+^ B-lymphocyte aggregates as indicated by maximum radial cell count. The molecular profiles of six *IL17A*^+^*/CD21L*^+^ and six *IL17A*^-^*/CD21L*^-^ synovia were determined by complementary DNA microarray analysis.

**Results:**

By RT-PCR, 26% of synovia expressed *IL17A* and 52% expressed *CD21L*. This provided the basis for distinguishing four subgroups of rheumatoid synovia: *IL17A*^+^*/CD21L*^+^ (18.5% of synovia), *IL17A*^+^*/CD21L*^-^ (7.5%), *IL17A*^-^*/CD21L*^+^ (33.3%) and *IL17A*^-^*/CD21L*^-^ (40.7%). While the subgroups did not predict clinical outcome measures, comparisons between the synovial subgroups revealed the *IL17A*^+^*/CD21L*^+^ subgroup had significantly larger CD20+ B-lymphocyte aggregates (*P* = 0.007) and a gene expression profile skewed toward B-cell- and antibody-mediated immunity. In contrast, genes associated with bone and cartilage remodelling were prominent in *IL17A*^-^*/CD21L*^-^ synovia.

**Conclusions:**

Rheumatoid synovia can be subdivided on the basis of *IL17A* and *CD21L* gene expression. Ensuing molecular subgroups do not predict clinical outcome for patients but highlight high inflammation and the predominance of B-lymphocyte mediated mechanisms operating in *IL17A*^+^*/CD21L*^+^ synovia. This may provide a rationale for more refined therapeutic selection due to the distinct molecular profiles associated with *IL17A*^+^*/CD21L*^+^ and *IL17A*^-^*/CD21L*^-^ rheumatoid synovia.

## Introduction

Rheumatoid arthritis (RA) is a chronic inflammatory disease that principally affects the synovial lining of joints. The associated synovial inflammation is heterogeneous encompassing histological features that distinguish fibroid, myeloid or lymphoid pathotypes, and associated molecular signatures [1, 2]. Where the inflammation is dominated by lymphocytes, cellular organisation covers a spectrum from a diffuse, less organised infiltrate, through increasing formation of lymphoid aggregates to more highly organised ectopic lymphoid follicle-like structures (ELS) with germinal centres [2, 3]

Mechanisms that underlie lymphocyte aggregation and ELS formation in rheumatoid synovium closely follow those involved in the formation of secondary lymphoid tissue during development. A source of lymphotoxin-β (LTβ) is crucial, which in inflamed synovium includes group 3 innate lymphoid cells (ILC3s) or lymphoid tissue inducer (LTi) cells, and probably also infiltrating B- and T-lymphocytes. LTβ activates pre-follicular dendritic cells (pre-FDCs; see below) and NFκB-inducing kinase-positive (NIK^+^) endothelial cells [4, 5] to produce chemokines and upregulate adhesion molecules. Key chemokines, including CXCL13 play crucial roles in the entry of B cells into the inflamed tissues that may further amplify the inflammatory process [6].

Mature FDCs are also a requisite for ELS neogenesis, particularly for the progression towards ELS with germinal centres (GC^+^ ELS), facilitating the production of high-affinity antibodies [7]. FDCs originate from platelet-derived growth factor-β-positive (PDGFRβ^+^)-perivascular pre-FDCs, which initially also express FDC-M1 (alternatively named milk fat globule epidermal growth factor 8; MFGE8) [4]. Mature FDCs are characterised by expression of the long isoform of the CD21 gene (*CD21L*) [8]. The formation of GC^+^ ELS in rheumatoid synovia favours the affinity maturation of B cells [9] and is thought to support the local production of the anti-cyclic citrullinated auto-antibodies associated with RA [7], although the true function of these synovial ELS is not clear. Known also as lymphoid follicles, ELS are present in 44–58% of rheumatoid synovial membranes with ~21–24% having GCs and CD21L^+^ FDC networks [10, 11]. There are associations between the presence of GC^+^ ELS in synovium, presence of rheumatoid factor (RF) and greater disease severity [11]. Such features demarcate rheumatoid inflammation that is more obviously B-lymphocyte driven.

As well as the key chemokines LTβ, CXCL13 and CXCL12, a number of cytokines also make well-defined contributions to the patterns of synovial inflammation seen in RA. Interleukin (IL)-17A is produced in a varied percentage of rheumatoid joint synovia [12, 13], with evidence suggesting that the presence of IL-17A is both predictive of disease progression in RA [12] and contributes to the inflammation by synergising with the actions of other key inflammatory mediators such as IL-1β, IL-6 and TNF-α [14, 15]. Meta-analysis of randomised controlled clinical trials for the treatment of RA with biological agents that neutralise IL-17A further emphasise the importance of the IL-17A pathway to RA [16].

Interleukin-17A is produced by a variety of cells including CD4^+^ Th17 cells, CD8^+^ T cells, natural killer cells, γδ-T cells, mast cells, and double-negative CD3^+^ T cells. In rheumatoid synovium, Th17 cells are found in lymphocyte-enriched areas [13] and in the vicinity of IL-23 producing monocytes [17] implicating the IL-17A/IL-23 axis in ELS formation [18, 19]. A key feature of Th17 cells is their plasticity, with different states achieved in humans distinguished by co-expression of the signature cytokine, IL-17A (and IL17F), alongside other cytokines like IL-10 or IFNγ [20–23]. In turn, the various combinations of cytokines impart distinct cellular effector functions. Consequently, Th17 cells function within a spectrum that spans from regulatory to more pathogenic, but not necessarily dependent on IL-17A production.

A key objective in the management of RA is the application of personalised therapy for control of joint synovial inflammation. In this study, we consider the expression of the *CD21L* and *IL17A* genes in rheumatoid synovium, reflecting the combined presence of FDCs and contribution from IL-17A to the synovial inflammation. We sought to determine whether expression of *IL17A* and *CD21L* in synovial tissue is associated with a distinct phase(s) of ELS neogenesis, and if the associated gene expression profile could aid the stratification of rheumatoid inflammation.

## Materials and methods

### Patients, synovial tissues and classification

All participants in this study gave written informed consent. The study was approved by the Multi-region Health and Disability Ethics committee (New Zealand), Ref No. MEC/06/02/003. Clinical data was obtained from medical record review.

Fifty-four synovia were obtained during joint replacement surgery from 45 patients with RA, as defined by the American Rheumatism Association 1987 classification criteria [24]. Multiple synovia were obtained from 8 patients, either at the same time or after periods of 5–67 months. From 3–6 resected pieces (≤0.4 cm^3^) of each synovium were stored frozen in liquid nitrogen, with each piece randomly assigned for various analyses of gene expression, as required. An additional 2–4 resected pieces were snap frozen embedded in tissue-tek for immuno-histology.

Total RNA (TRNA) was extracted from ~50–100 mg of synovial tissue and reverse transcribed as previously described [17]. Using the following sense and antisense primers (respectively) for *IL17A*: 5’-ATG ACT CCT GGG AAG ACC TCA TTG-3’ and 5’-TTA GGC CAC ATG GTG GAC AAT CGG-3’; and *CD21L*: 5’-GTG GAT TTA CTT TGA AGG GCA-3 and 5’-GGC ATG TTT CTT CAC ACC G-3’, the expression of *IL17A* and *CD21L* genes was assessed by PCR and agarose gel-based detection. On this basis synovia were classified as positive or negative for *IL17A* and *CD21L* expression and assigned to one of four groups.

### Assays of gene expression

Levels of gene expression were further quantitated by standard real-time PCR (qRT-PCR) or by digital PCR (dPCR) assays using commercially available *IL17A* (Hs00174383_m1), GAPDH (Hs99999905_m1) Taqman assays (Applied Biosystems) and a custom-designed *CD21L* Taqman assay based on the reporter sequence within CD21L: 5’-ACGGTGTGAAGAAACAT-3’ (Applied Biosystems). For qRT-PCR, the analysis of each gene was performed in triplicate, with comparisons relative to tonsil standard RNA (ng) and the results for individual samples expressed as the mean for each gene relative to the mean of GAPDH RNA. Digital-PCR analysis was performed as previously described [25], using Quantstudio 3D digital PCR 20K chip kits and utilising a single chip per sample. Digital results are expressed as absolute values (i.e. non-normalised) for the number of gene specific RNA molecules per ng of RNA.

### Synovial immunohistology

For assessment of lymphoid aggregation 7μm cryostat sections from replicate synovial tissue samples were stained with Gill’s haematoxylin 3 and 0.5% alcoholic eosin. Samples were de-identified and the size of lymphoid aggregates quantified by maximum radial cell count (MRCC) as previously described [26].

Consecutive sections were immunohistochemically stained as previously described [17] for the expression CD21L (anti-CD21L; Santa Cruz Biotechnology, Inc.) or T- and B-lymphocytes (anti-CD3 and anti-CD20 respectively; DakoCytomation) using mouse monoclonal antibodies. Non-specific antibody binding was blocked by incubating sections with 2.5% normal rabbit serum (Sigma). Primary antibodies were detected with rabbit anti-mouse IgG-conjugated horse radish peroxidase (HRP; DakoCytomation) visualised with chromogenic substrate (DAB, 1 mg/ml; DAKO Corporation), and nuclei counter-stained with Gill’s haematoxylin 3. Photomicrographs were taken using an Olympus BX50 microscope fitted with Spot RT digital camera and software (Diagnostic Instruments).

Values are expressed as group median and the interquartile range (IQR) unless otherwise stated. Differences in gene expression levels, MRCC and aggregate numbers among synovial subgroups were determined using the Kruskal-Wallis test, followed by paired comparisons with Dunn’s Multiple Comparison test. Multivariate analysis was performed for CD21L/IL17A subtype associations with disease characteristics, aggregate and gene expression using generalised estimating equation population-averaged model analyses (Log Binomial and Modified Poisson Regression with exchangeable correlations) in MedCalc v11.4.2.0. All other statistical analyses were performed using Prism 4 for Windows v4.03 (GraphPad Software). Values of *P* < 0.05 were considered statistically significant.

### Microarray analysis

For microarray analysis a subset of 12 rheumatoid synovia, classified on the basis of gene expression as *IL17A*^+^*/CD21L*^+^ (n = 6) or *IL17A*^-^*/CD21L*^-^ (n = 6), obtained from 10 patients were identified. Two *IL17A*^+^*/CD21L*^+^ synovia (P1-1 and P1-2) were obtained from one patient, 14-months apart. A second patient provided one *IL17A*^+^*/CD21L*^+^ synovium (P4-1) and 5-months later, one *IL17A*^-^*/CD21L*^-^ synovium (P4-2).

The purity and integrity of extracted synovial TRNA were established by capillary electrophoresis (Bioanalyser; Agilent). Sample hybridisation and microarray data analysis was performed by the Otago Genomics Facility (University of Otago, Dunedin). Briefly, 5 μg total RNA was amplified and labelled using the MessageAmp^™^ Premier RNA Amplification Kit (Ambion), according to the manufacturer’s specifications. Ten μg of biotinylated complementary RNA was then fragmented and hybridised at 45°C for 16 h to GeneChip Human Genome U133 Plus 2.0 arrays containing ~38,500 characterised genes (~54,000 probe sets; Affymetrix).

To compare gene expression profiles from the different arrays, data was analysed with the Affymetrix Expression Console version 1.1 (MAS 5.0) using Affymetrix default analysis settings and with Robust Multiarray Average (RMA) as the normalisation method [27]. Raw and normalised data (GEO accession: GSE38064) are available online at http://www.ncbi.nlm.nih.gov/geo/). Differences in gene expression were calculated as fold-changes by comparing the mean of normalised values for the six *IL17A*^+^*/CD21L*^+^ synovia with the mean of normalised values for the six *IL17A*^-^*/CD21L*^-^ synovia, for each probe set. Significance was determined by generating a t-statistic and p-value for each probe set using Bioconducter Software AffyImGUI [28]. A two-sided *P* < 0.05 was considered statistically significant.

To identify genes with heterogeneous or related expression profiles, hierarchical cluster analysis was applied. Normalised signals for each probe set with significantly different expression were median-centred and analysed by complete-linkage hierarchical clustering of genes and arrays using Gene Cluster and visualised in TreeView (online at http://rana.lbl.gov/EisenSoftware.htm) [29].

To determine the pathways and biological processes represented by the genes with significantly different levels of expression in *IL17A*^+^*/CD21L*^+^ synovia compared to *IL17A*^-^*/CD21L*^-^ synovia, gene ontology analysis was performed using the Protein ANalysis Through Evolutionary Relationships database (PANTHER; online at http://panthedb.org) [30]. Statistically significant over-representation of genes involved in various pathways and processes was determined by comparing the genes with significantly different expression against a reference *Homo sapiens* NCBI gene list using the binomial statistic [31]. Statistical significance was considered as *P* < 0.05.

## Results

### Patients and synovial tissues

Our patient cohort comprised 45 patients with RA providing 54 synovia. Mean ± SE age of this cohort was 61 ± 1.7 years, with mean ± SE disease duration 9.8 ± 1.4 years. Sixteen (36%) patients were male, 31 (69%) had nodules and 42 (93%) had radiographic erosions. Multiple synovia were obtained from 8 of 45 patients, with 4 patients each providing 2 separate synovia at different times, 5–67 months apart. A further 4 patients, each provided two separate synovial samples from two different joints at the same time; one of these patients provided an additional synovium ~12 months later.

### *IL17A* and *CD21L* gene expression distinguishes subgroups of rheumatoid synovia

Rheumatoid synovia (n = 54) were assessed for *IL17A* and *CD21L* gene expression using PCR and agarose gel-based detection, thereby classifying synovia within one of four possible subgroups (*IL17A*^+^*/CD21L*^+^, *IL17A*^+^*/CD21L*^-^, *IL17A*^-^*/CD21L*^+^, or *IL17A*^-^*/CD21L*^-^). Applying this approach, we found that 14 (26%) of 54 rheumatoid synovia, had detectable *IL17A* gene expression and 28 (52%) *CD21L* expression. Considering the four possibilities for expression of these two genes, 10 of 54 synovia (19%) were identified as *IL17A*^+^*/CD21L*^+^, and 22 synovia (41%), identified as *IL17A*^-^*/CD21*^-^. Synovia with *IL17A* expression alone were comparatively rare (7% in this cohort) compared to those with *CD21L* expression alone (33%). Demographics of patients contributing the IL17A and CD21L classified synovia are shown in Table 1.

**Table 1:** Patient demographic and clinical data for synovia classified by the expression of *IL17A* and *CD21L*.

	*IL17A*^+^*/CD21L*^+^	*IL17A*^+^*/CD21L*^-^	*IL17A*^-^*/CD21L*^+^	*IL17A*^-^*/CD21L*^-^
No. of synovia	10 (19%)	4 (7%)	18 (33%)	22 (41%)
No. of Patients^a^	9	4	17	20
Age, median (IQR) years	62.5 (36.5–68.5)	66.5 (58.5–70.5)	65.5 (46.0–69.5)	64 (54.5–70.0)
% Female	56%	100%	59%	90%
RF Positive	8	4	17	19
ACPA Positive	6/8^b^	3/4	12/15	12/13
Subcutaneous nodules present	6	3	10	12
Radiographic erosions present	8	4	17	17
Disease duration, median (IQR) years	10.5 (4.8–23.0)	8.0 (5.3–14.6)	16.1 (8.0–26.0)	16.5 (8.0–24.0)
ESR, median (IQR) mm/hr	27.5 (19.5–40.0)^c^	36.5 (N/A)	25.0 (13.5–50.0)	26.5 (15.0–44.5)
CRP, median (IQR) mg/dL	12.0 (4.0–16.0)^c^	31.5 (13.5–56.5)	7.0 (4.0–18.5)	19.5 (4.0–14.0)
Taking DMARDs	9	4	17	17

^a^Patient cohort (n = 45) included 8 patients providing multiple (2–3) synovia. Following tissue classification, 5 patients contributed to more than one classification group.

^b^ACPA data was only available for 37/45 patients with 84% of 37 patients, ACPA^+^. ACPA positive data show ACPA^+^ patients per number of patients tested within each classification group.

^c^ESR and CRP data was only available for 33/45 patients (73%).

The IQR could not be determined for subgroups of low samples size, denoted by N/A.

Abbreviations: RF, rheumatoid factor; ACPA, anti-citrullinated peptide antibodies; ESR, erythrocyte sedimentation rate; CRP, C-reactive protein; DMARDs, disease modifying anti-rheumatic drugs. No patients were receiving biologic therapy.

We sought verification of a PCR-based approach to classifying synovia from the quantitation of transcript by real-time qRT-PCR. In all *IL17A*^+^ synovia (regardless of *CD21L* expression), median expression level of *IL17A* was 0.077 ng (IQR = 0.04–0.18 ng); In *IL17A*^-^ synovia, no quantifiable transcript was detected. In all *CD21L*^+^ synovia (regardless of *IL17A* expression), median expression of *CD21L* was 0.011 ng (IQR = 0.006–0.041 ng). In addition, low levels of *CD21L* transcript (median expression 0.0015 ng; IQR = 0.0004–0.0028 ng) were detected by real-time qRT-PCR in 23/26 (88%) *CD21L*^-^ synovia. The lack of overlap in the IQR for transcripts in *IL17A*^+^ *vs IL-17A*^-^ synovia or *CD21L*^+^ *vs CD21L*^-^ synovia indicated the use of PCR and gel-based assays to distinguish *IL17A/CD21L* synovial subgroups. Median expression for both *IL17A* and *CD21L* genes, amongst the four possible synovial subgroups, is summarised in Table 2. These data establish significant differences in *IL17A* and *CD21L* expression, particularly between the *IL17A*^+^*/CD21L*^+^ and *IL17A*^-^*/CD21L*^-^ synovia.

**Table 2:** Quantitative gene expression in synovial tissue subgroups defined by the expression of *IL17A* and *CD21L*.

Synovial Subgroup	Number (%)	*IL17A* expression	*CD21L* expression
*IL17A*^+^/*CD21L*^+^	10 (18.5)	0.095 (0.073–0.176)^a^	0.011 (0.003–0.077)
*IL17A*^+^/*CD21L*^-^	4 (7.5)	0.086 (0.033–0.713)	0.005 (0.002–0.008)
*IL17A*^-^/*CD21L*^+^	18 (33.3)	0 (0–0.030)^†^^,^^‡^	0.005 (0.002–0.023)
*IL17A*^-^/*CD21L*^-^	22 (40.7)	0 (0–0)^†^^,^^§^	0.002 (0–0.006)*
*P*-value		<0.0001^b^	0.030

Synovial tissues were classified into subgroups based on PCR-based gel assays for *IL17A* and/or *CD21L* expression.

^a^Values from qRT-PCR are presented as median expression in ng RNA relative to GAPDH, with IQR shown in parenthesis.

^b^Comparison among all *IL17A*/*CD21L* subgroups was performed by Kruskal-Wallis test, followed by paired comparisons with Dunn’s Multiple Comparison test.

*P<0.05,

^†^P<0.001 compared to *IL17A*^+^/*CD21L*^+^ synovia.

^‡^P<0.05,

^§^P<0.01 compare to *IL17A*^+^/*CD21L*^-^ synovia.

**Fig 1:**
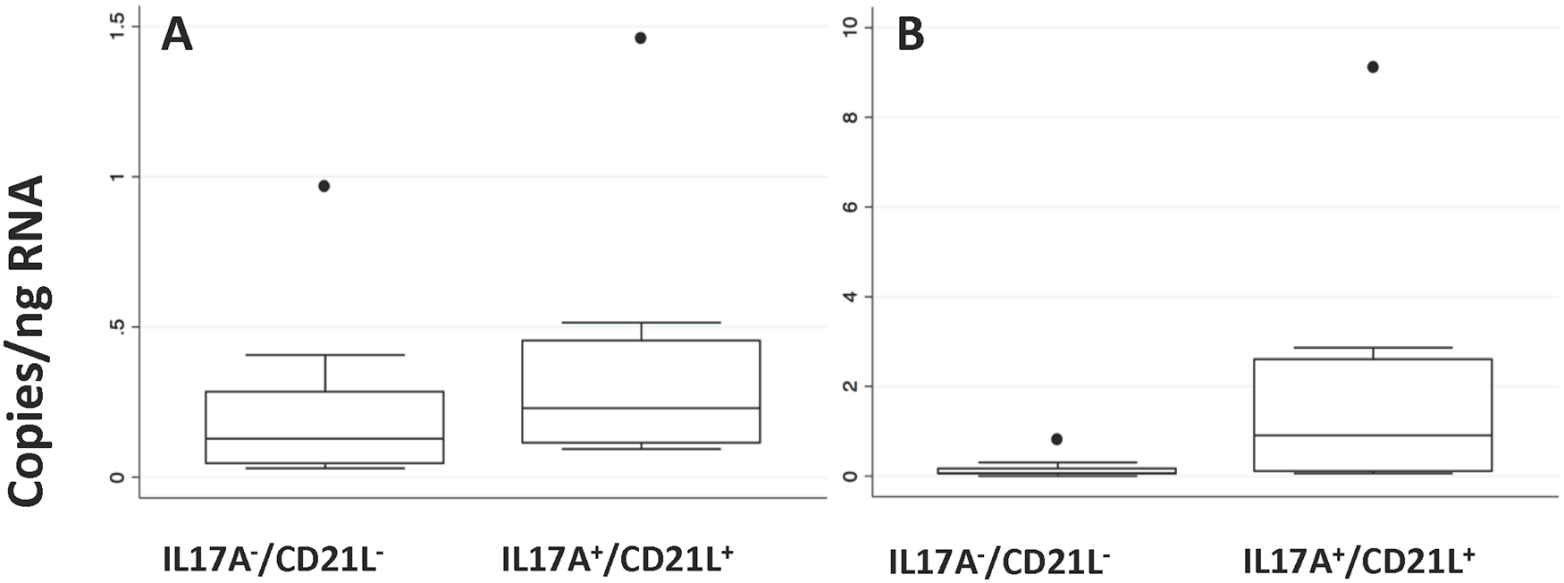
Absolute measures of synovial *IL17A* or *CD21L* gene expression. Synovia were originally classified into *IL17A*^-^/*CD21L*^-^ (n = 12) or *IL17A*^+^/*CD21L*^+^ (n = 10) subgroups. Digital PCR was used to establish absolute measures of (A) *IL17A* expression or (B) *CD21L* expression in each of these subgroups. Outliers within each subgroup are shown as individual dots (●).

We reasoned that absolute (i.e. non-normalised) measures of *IL17A and CD21L* expression, obtained using dPCR, would be compatible with PCR and gel-based detection, and might provide universally comparable measures for distinguishing between synovial subgroups. We compared absolute measures of *IL17A* and *CD21L* expression between *IL17A*^-^/*CD21L*^-^ and *IL17A*^+^/*CD21L*^+^ synovia (Fig 1). The more sensitive dPCR assays reveal few synovia with a complete lack of gene expression but data highlight the skew towards higher absolute measures of *IL17A* and *CD21L* expression in *IL17A*^+^/*CD21L*^+^ synovia. Our data suggest absolute measures of expression ≥ 0.25 copies/ng RNA for *IL17A* (Odds ratio (OR) = 2.4) and ≥ 0.7 copies/ng RNA for *CD21L* (OR = 11), as guidelines for distinguishing “positive” from “negative” expression.

### Combined *IL17A* and *CD21L* gene expression is associated with increased synovial lymphocyte aggregation

Individually, IL-17A and CD21L have been implicated in lymphoid neogenesis [19, 32]. Therefore, we sought evidence for an association between combined *IL17A* and *CD21L* gene expression and lymphoid organisation in rheumatoid synovia. A subset of synovia (n = 30/54 synovia; 26 patients) comprising 27% *IL17A*^+^*/CD21L*^+^, 10% *IL17A*^+^*/CD21L*^-^, 23% *IL17A*^-^*/CD21L*^+^ and 40% *IL17A*^-^*/CD21L*^-^ were examined histologically. Prominent inflammation and more obvious organisation of infiltrating lymphocytes into aggregates was a feature of *IL17A*^+^*/CD21L*^+^ synovia (data not shown). In comparison, *IL17A*^-^*/CD21L*^-^ synovia had comparatively fewer inflammatory cells and demonstrated a largely diffuse inflammatory infiltrate. A mixed pattern was observed in *IL17A*^+^*/CD21L*^-^ and *IL17A*^-^*/CD21L*^+^ synovia that included some areas of diffuse infiltration and, particularly in perivascular locations, some organisation of inflammatory cells into aggregates.

**Fig 2:**
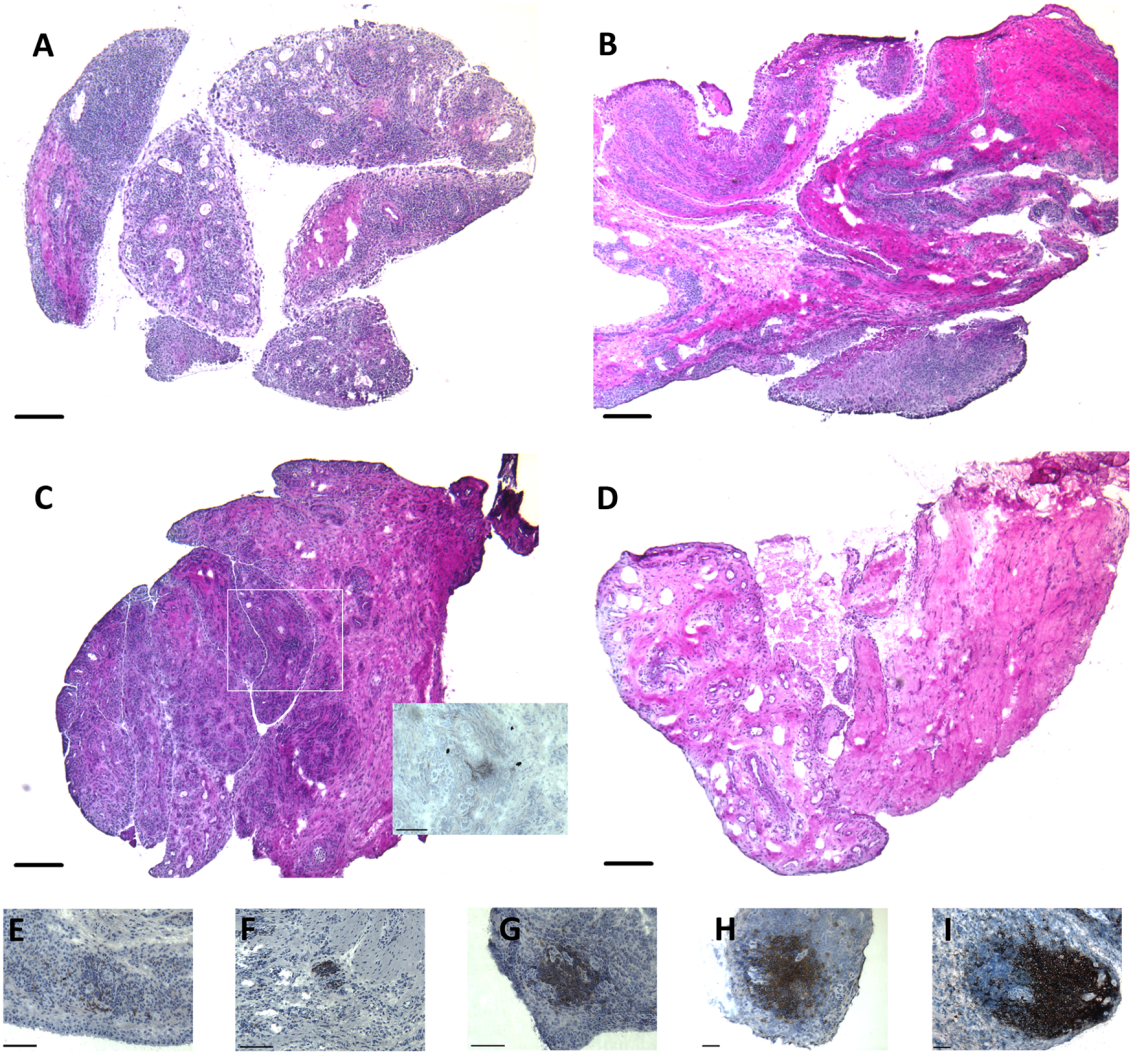
Appearance and composition of synovial lymphoid aggregates. Classified synovia show differences in tissue histology, exemplified in sections from synovia classified as (A) *IL17A*^+^/*CD21L*^+^, (B) *IL17A*^+^/*CD21*-, (C) *IL17A*^-^/*CD21L*^+^ and (D) *IL17A*^-^/*CD21L*^-^ (all stained with haematoxylin and eosin). Aggregates of CD20^+^ B-lymphocytes (brown staining) within synovia cover a spectrum, including smaller, less obvious clusters (insert to Panel C; Panel E) through those of varying size and more obvious levels of organisation as exemplified in panels F-I. Larger lymphoid aggregates were more regularly seen in *IL17A*^+^/*CD21L*^+^ synovia. Scale bar: 200 μm (A-D), 100 μm (E-G), and 50 μm ((H-I).

We next assessed the relationship between expression of *IL17A* and *CD21L* and size of aggregates containing CD20^+^ B-lymphocytes. As indicated by MRCC, the median size of aggregates containing CD20^+^ B-lymphocytes was significantly different between the synovial subgroups (*P* = 0.007; Table 3). Post-hoc analysis identified significantly larger, but not more, aggregates containing CD20^+^ B-lymphocytes within *IL17A*^+^*/CD21L*^+^ synovia, compared to those in *IL17A*^-^*/CD21L*^+^ synovia and *IL17A*^-^*/CD21L*^-^ synovia (*P* < 0.05; Table 3). Together these results suggest that the combined expression of *CD21L* and *IL17A* genes is associated with the presence of larger aggregates containing CD20^+^ B-lymphocytes in rheumatoid synovia. Notably the larger aggregates showed segregation of CD20^+^ B- and CD3^+^ T-lymphocytes into distinct regions (Fig 2).

**Table 3:** The MRCC and number of CD20^+^ B-lymphocyte aggregates among synovial subgroups.

Synovial Subgroup	MRCC (Cells)	Number (Aggregates)
*IL17A*^+^/*CD21L*^+^	7.5 (5.5–13.5)^a^	3 (0.5–6.5)
*IL17A*^+^/*CD21L*^-^	5 (4–6)	2 (N/A)
*IL17A*^-^/*CD21L*^+^	5 (0–7)*	1 (0–2)
*IL17A*^-^/*CD21L*^-^	5 (3–7)*	1 (0–3.5)
*P*-value	0.007^b^	0.54

^a^Values are presented as median cells or aggregates, as appropriate, with IQR shown in parenthesis.

^b^Comparison among all *IL17A*/*CD21L* synovial subgroups was performed by Kruskal-Wallis test, followed by paired comparisons with Dunn’s Multiple Comparison test.

*P<0.05, compared to *IL17A*^+^/*CD21L*^+^ synovia. The IQR could not be determined for subgroups of low sample size, denoted by N/A.

### Combined *IL17A* and *CD21L* expression is not associated with clinical outcomes

We assessed whether the combined expression of *IL17A* and *CD21L* genes was associated with standard clinical features and outcomes for RA. We compared disease characteristics, including age at onset, disease duration, and presence of erosions and subcutaneous nodules; measures of erythrocyte sedimentation rate (ESR), serum C-reactive protein (CRP), rheumatoid factor (RF) and anti-citrullinated peptide antibody (ACPA); measures of disease severity including van der Heijde Sharp score [33], number of previous joint surgeries; measures of disease impact including change in HAQ score, number of joint injections and hospital admissions per year; as well as number of disease modifying anti-rheumatic medications used since diagnosis. There was no significant difference in any of these variables between patients separated according to *CD21L*/*IL17A* synovial subgroup classification (data not shown).

Multi-variate analysis showed no association between the size of aggregates containing CD20^+^ B-lymphocytes and disease outcome, reflected by Sharp scores or as Sharp scores per year(s) of disease duration.

### Distinctive molecular profiles are associated with *IL17A* and *CD21L* expression in rheumatoid synovia

While the *IL17A*/*CD21L* synovial subsets did not associate with clinical outcomes we considered that expression of the two genes may be associated with distinct molecular profiles. We anticipated such a distinction might provide clinically relevant insight into the heterogeneity of RA and potentially aid therapeutic selection.

**Fig 3:**
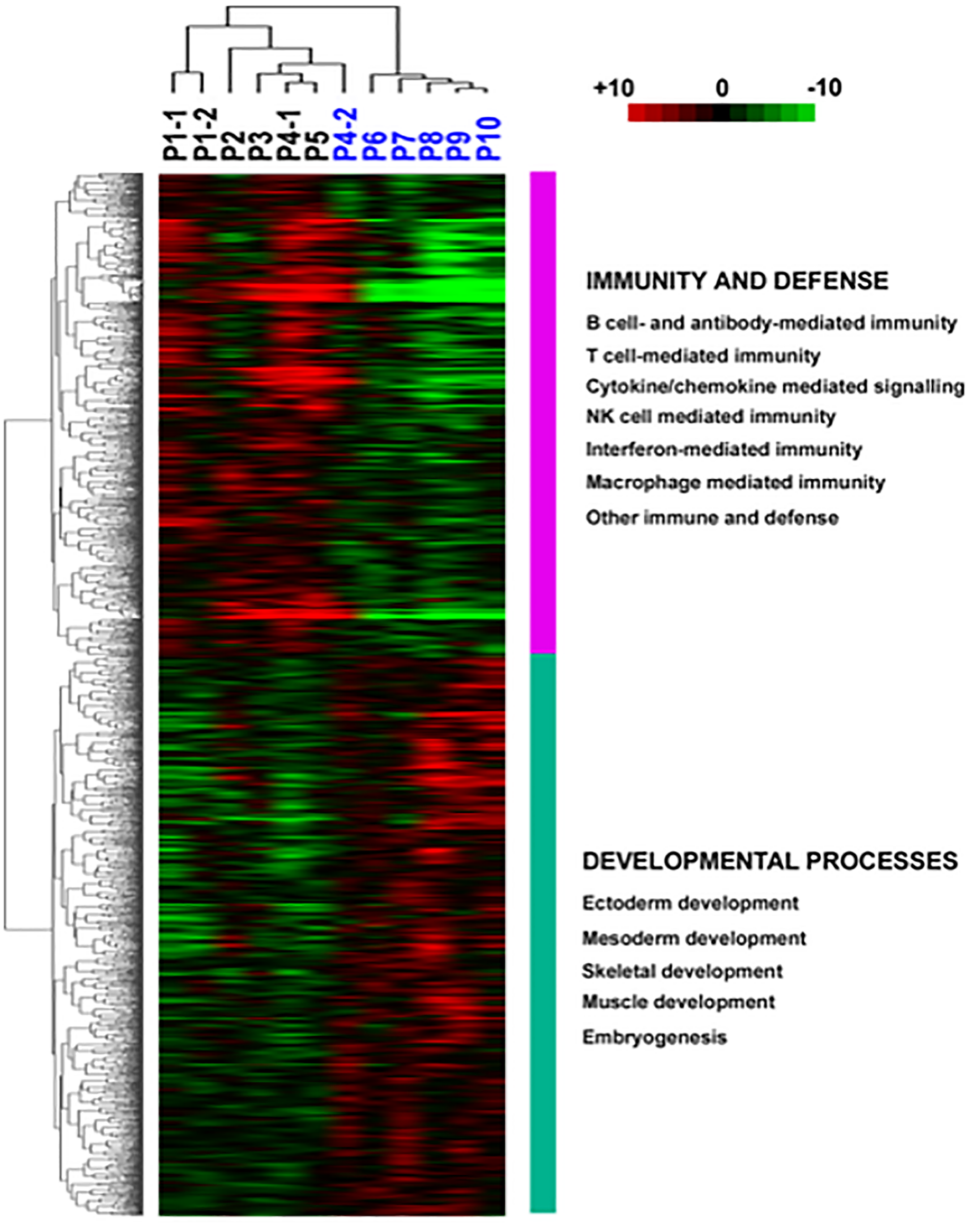
Hierarchical cluster analysis of rheumatoid synovia. Shown are synovia ordered by hierarchical clustering of 3,092 transcripts with significant differential expression between IL17A^+^/CD21L^+^ (black) and IL17A^-^/CD21L^-^ (blue) synovial subgroups. Paired synovia P1-1 and P1-2 are from one individual at the same time, and paired synovia P4-1 and P4-2 from another individual, 5 months apart. The matrix shows genes with significantly different expression in rows relative to the individual synovia samples in columns. Red indicates higher than median expression (black) and green lower than median expression across all assessed tissues. Coloured vertical bars indicate clusters of differentially expressed genes classified by PANTHER as “Immunity and defense”, and “Developmental processes”.

Gene expression profiling of six *IL17A*^+^*/CD21L*^+^ and six *IL17A*^-^*/CD21L*^-^ synovia identified 3,092 transcripts with significantly different expression levels. Of these 1,433 transcripts were up-regulated in *IL17A*^+^*/CD21L*^+^ synovia and 1,659 in *IL17A*^-^*/CD21L*^-^ synovia. Hierarchical cluster analysis of the molecular profiles grouped the synovia into their corresponding *IL17A*^+^*/CD21L*^+^ and *IL17A*^-^*/CD21L*^-^ subgroups (Fig 3). The *IL17A*^+^*/CD21L*^+^ synovia show greater variation in their molecular profiles, compared to *IL17A*^-^*/CD21L*^-^ synovia (Fig 3). Two separate synovia from the same patient, obtained 14 months apart and both originally classified as *IL17A*^+^*/CD21L*^+^, subsequently clustered with other *IL17A*^+^*/CD21L*^+^ synovia. In addition, two separate synovia from a second patient, which originally classified into different *IL17A/CD21L* subgroups, clustered appropriately; the *IL17A*^-^*/CD21L*^-^ synovium was obtained five months before the *IL17A*^+^*/CD21L*^+^ synovium (Fig 3). Thus, the gene expression profiles of synovia from the same *IL17A/CD21L* subgroup are similar between different patients. Moreover, the gene expression profiles are different between synovia of different *IL17A/CD21L* subgroups obtained from the same patient.

### High inflammatory activity in *IL17A*^+^*/CD21L*^+^ synovial molecular profiles

Gene ontology analysis was performed to further characterise the genes up-regulated in *IL17A*^+^*/CD21L*^+^ or *IL17A*^-^*/CD21L*^-^ synovia. Using the PANTHER database (Thomas, 2003), this analysis incorporated 1,222 (85.3%) of the 1,433 genes significantly up-regulated in *IL17A*^+^*/CD21L*^+^ synovia and 1,451 (87.5%) of the 1,659 genes significantly up-regulated in *IL17A*^-^*/CD21L*^-^ synovia.

Genes up-regulated in *IL17A*^+^*/CD21L*^+^ were significantly over-represented in 57 categories of biological processes. Of these the most significant was “Immunity and defence” (*P* = 2.31E-11). Within this category are a number of well-defined processes that were also significantly over-represented by genes with up-regulated expression in *IL17A*^+^*/CD21L*^+^ synovia (Fig 3). These include “B-cell- and antibody-mediated immune processes” with associated genes that are specifically involved in B-cell receptor (BCR) formation and expression (e.g. *IGHM*, *IGK*, *IGL*, *CD19*, *CD79A*, *CD79B*), BCR signal modulation and transduction (e.g. *BLNK*, *PLCG2*, *VAV-2 MAPK1*, *MAPK14*, *FCRL5*) and immunoglobulin class-switching from IgM (e.g. *IGHA*, *IGHD*, *IGHG*). In addition, genes associated with lymphocyte co-stimulation (e.g. *ICOSLG*, *CTLA-4*, *SLAMF3*, *SLAMF7*) and antigen presentation (e.g. *HLA-DOA*, *HLA-DOB*, *HLA-C*) were up-regulated in *IL17A*^+^*/CD21L*^+^ synovia. These results indicate that B-lymphocytes and other antigen-presenting cells are present and that there is a substantial B-lymphocyte focused, immune component to the inflammation associated with *IL17A*^+^*/CD21L*^+^ synovia. Interestingly, amongst the significantly under-represented biological processes from genes up-regulated in *IL17A*^+^*/CD21L*^+^ synovia were “protein biosynthesis” (*P* = 9.87E-04) and “mitosis” (*P* = 0.032)”.

### Bone and cartilage remodelling in *IL17A*^-^*/CD21L*^-^ synovial molecular profiles

Fifty-six categories of biological processes were up-regulated in *IL17A*^-^*/CD21L*^-^ synovia. Microarray data revealed that the most over-represented category was “Developmental processes” (*P* = 1.33E-09). This category comprised a number of well-defined processes contributing to inflammation and tissue remodelling (Fig 3). Over-represented genes include those associated with synovial hyperplasia (e.g. *EGFR*, *FGF18*, *FGFR1*, *SOCS4*, *SOCS7*), bone and cartilage morphogenesis (e.g. *COL1A2*, *OPG*, *SCUBE2*, *BGN*, *SHOX2*, *SOX9*), and cell migration and adhesion (e.g. *SLIT3*, *ROBO2*, *RHOA*, *PCDHB2*, *PCDHGB7*, *CDH2*, *CDH19*, *YES1*). These results highlight that significant tissue remodelling, promoted through the activities of synovial fibroblasts, chondrocytes, osteoblasts and osteoclasts, occurs in association with any inflammation in *IL17A*^-^*/CD21L*^-^ synovia.

### Pathways over-represented in the synovial subgroups

PANTHER classification was utilised to determine relevant and distinct pathways between the synovial *IL17A/CD21L* subgroups [30]. This analysis accommodated 767 of the genes (53.5%) significantly up-regulated in *IL17A*^+^*/CD21L*^+^ synovia and 1,004 of the genes (60.5%) significantly up-regulated in *IL17A*^-^*/CD21L*^-^ synovia.

Among the 29 pathways identified from genes in *IL17A*^+^*/CD21L*^+^ synovia were a number of inflammatory signalling pathways (Table 4). Consistent with the analysis of biological processes, B- and T-lymphocyte activation pathways were significantly over-represented. In addition, TLR, integrin, G-protein, and cytokine- and chemokine-mediated signalling pathways, likely to contribute to the activation and trafficking of inflammatory cells were up-regulated (Table 4).

**Table 4:** Pathway gene ontology analysis of genes significantly up-regulated in *IL17A*^+^*/CD21L*^+^ and *IL17A*^-^*/CD21L*^-^ rheumatoid synovia.

PANTHER pathway	P-value
**Up-regulated *IL-17A***^**+**^***/CD21L***^**+**^	
Apoptosis signalling	1.17E-09
Heterotrimeric G-protein signalling Gqα and Goα	2.33E-07
B cell activation	8.57E-06
Toll like receptor signalling	1.61E-04
Interleukin signalling	3.40E-04
Heterotrimeric G-protein signalling Giα and Gsα	4.06E-04
Inflammation mediated by chemokine and cytokine	4.52E-04
Integrin signalling	0.0018
Axon guidance mediated semaphorins	0.0047
VEGF signalling	0.0057
**Up-regulated *IL-17A***^**-**^***/CD21L***^**-**^	
Axon guidance by Slit/Robo	1.53E-04
Alzheimer disease-amyloid secretase	0.0036
Thyrotropin-releasing hormone receptor signalling	0.014
Gamma-aminobutyric acid synthesis	0.016
Alzheimer disease-presenilin	0.017
Cadherin signalling	0.020
Inflammation mediated by chemokine and cytokine signalling	0.026
Heterotrimeric G-protein signalling rod outer segment	0.028
Oxytocin receptor mediated signalling	0.049

The top 10 pathways significantly over-represented by up-regulated genes in *IL-17A*^+^*/CD21L*^+^ or *IL-17A*^-^*/CD21L*^-^ synovia are shown. P < 0.05 considered statistically significant.

By comparison only nine pathways were over-represented by genes significantly upregulated in *IL17A*^-^*/CD21L*^-^ synovia (Table 4). These pathways included cadherin and cytokine- and chemokine-mediated inflammation signalling pathways. Notably the latter pathway featured suppressor of cytokine signalling 7 (*SOCS7*), which inhibits the expression of IL-23 and the Th17 cell transcription factor, retinoid-related orphan receptor-C (*RORC*) [34], potentially contributing to the absence of *IL17A* expression in *IL17A*^-^*/CD21L*^-^ synovia.

### Links between B-lymphocyte aggregation and *RGS13* expression

Finally, from microarray data, we sought evidence to explain the mechanism by which IL-17A and *CD21L*^+^ FDCs might contribute to the greater B-lymphocyte aggregation found in *IL17A*^+^/*CD21L*^+^ synovia. We considered genes associated with B-lymphocyte survival, co-stimulation and trafficking for separate analysis, including *IL23A*, *TLR9A*, *BAFF*, *APRIL*, *RGS13*, *RGS16* and *SIPA-1*.

**Fig 4:**
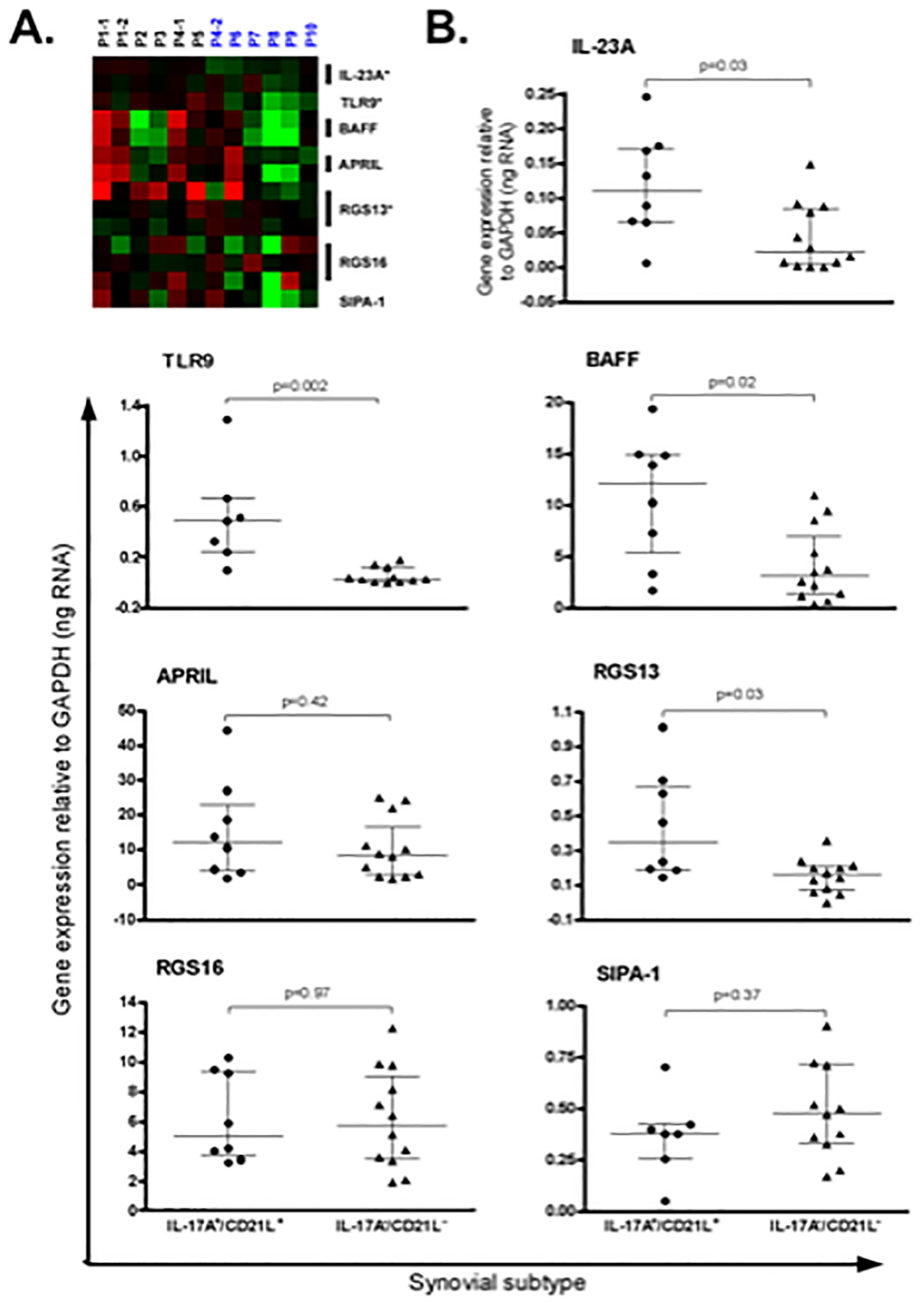
Gene expression in synovial subtypes. Expression of selected genes associated with B-lymphocyte survival, co-stimulation and trafficking was compared. Microarray data (A) shows genes in rows relative to the individual synovial samples with higher (red) or lower (green) median expression across all tissues indicated. Array data is clustered within *IL-17A*^+^/*CD21L*^+^ (black) or *IL17A*^-^/*CD21L*^-^ (blue) synovial subtypes showing greater expression (asterisks) of *IL-23A*, *TLR9A*, and *RGS13* in *IL-17A*^+^/*CD21L*^+^ synovia, when compared to *IL-17A*^-^/*CD21L*^-^ synovia. (B) Significantly greater expression of *IL23A*, *TLR9A*, *RGS13* and *BAFF* expression in *IL-17A*^+^/*CD21L*^+^ synovia was confirmed by qRT-PCR.

Microarray data indicated greater expression of *IL23A*, *TLR9A*, and *RGS13* in *IL17A*^+^/*CD21L*^+^ synovia, when compared to *IL17A*^-^/*CD21L*^-^ synovia (Fig 4). These patterns of expression were confirmed by qRT-PCR, alongside significantly greater expression of *BAFF*, in *IL17A*^+^/*CD21L*^+^ synovia (Fig 4). There was no significant difference in *RGS16* expression between synovial subtypes, but generally greater *RGS16* expression (by ~10-fold) compared to *RGS13*. Similarly, *APRIL* expression was not significantly different between synovial subgroups. While *APRIL* and *BAFF* were expressed at comparable levels in *IL17A*^+^/*CD21L*^+^ synovia, significantly less *BAFF* expression, (by ~4-fold) was evident in *IL17A*^-^/*CD21L*^-^ synovia. While not significant, there was a trend for more *SIPA1* transcript in *IL17A*^-^/*CD21L*^-^ synovia (Fig 4).

## Discussion

Variability of the inflammation involving synovial tissue is a feature of RA. Histologically, distinct types of synovial pathology are recognised, predominantly involving either monocyte/macrophages (myeloid pathotype), or lymphocytes (lymphoid pathotype), or alternatively with fibroblast involvement and comparatively less inflammation (fibroid pathotype) [35]. A key objective has been to utilise these differences in synovial inflammation to divide RA into clinically meaningful subgroups that might represent variation in the underlying pathology for individual patients. On this basis, it may be possible to predict disease outcome and/or determine optimal choice of therapy. Critical to this objective are approaches that accurately reflect the heterogeneity of inflammation and that distinguish different patterns of active immune-mediated disease.

In this study, we utilised expression of the *CD21L* gene as a molecular biomarker for FDCs which, when combined with measures of *IL17A* expression, formed the basis for classifying synovial tissues. Further we utilised digital-PCR to establish absolute measures of *CD21L* and *IL17A* gene expression that provide guidelines for international comparisons between tissues, regardless of assay.

Previous studies have identified FDCs amongst requisites for the development of the ectopic GC^+^ ELS observed in rheumatoid synovia [32]. Expression of *CD21L* reflecting these cells was measurable in most (~94%) synovia by qRT-PCR and dPCR, but generally at low levels. In practice, we found that application of guidelines for absolute measures of gene expression or the comparatively less sensitive agarose gel-based PCR assays introduce a practical working threshold that discriminate synovia as *CD21L*^+^ or *CD21*^-^. Synovia with the higher levels of *CD21L* expression featured well-organised ELS, with well-demarcated B- and T-cell segregation. However, GC^+^ ELS, and histologically distinguishable FDCs, were rare among our synovial samples. This high prevalence but low levels of *CD21L* gene expression and the relative paucity of GC^+^ ELS suggest that synovial *CD21L* expression reflects a tissue microenvironment capable, through the presence of FDCs (or their precursors), of developing ELS, rather than one where ELS are actually present.

There is now considerable experimental evidence to support the contribution of IL-17A to the pathogenesis of RA, varying from the linkage of Th17 cells with bone resorption [36] to observations of these same cells within and adjacent to synovial ELS [13]. However, a function directly attributable to IL-17A remains somewhat controversial, with other Th17 cell-derived cytokines also associated with the presence of synovial ELS [18]. Overall, we found *IL17A* gene expression in ~40% of synovia and that alone, *IL17A* gene expression was associated with larger lymphocytic aggregates in synovium. However, the combined expression of the *IL17A* and *CD21L* genes provided a clear distinction between different synovial subgroups, with *IL17A*^+^/*CD21L*^+^ synovia having significantly larger sized aggregates of lymphocytes that included CD20^+^ B-cells. Concomitantly there were no greater numbers of these B-cell containing aggregates, implicating a role for IL-17A in the stabilisation and/or expansion, but not necessarily the initiation of lymphoid aggregation. There is precedence for this in the murine system whereby continual IL-17A signalling is required to overcome a dissipating action of IL-23 and thereby maintain lymphoid aggregates [19, 37]. The combined data suggest that IL-17A signalling is important to the expansion and/or stability of B-cell and T-cell aggregates within rheumatoid synovia. The implication is that in synovia where IL-23 is also produced, low levels or a complete absence of IL-17A is likely to favour aggregate dissipation [19]. We observed greater *IL23A* gene expression in *IL17A*^+^/*CD21L*^+^ synovia that appears contrary to this process, but which is consistent with earlier reports [18]. However, this might reflect the threshold levels of IL-23 required to support Th17 cells and their production of IL-17A [38] as well as the contribution from multiple other cytokines [34]. Consequently, dual roles for IL-23 in promoting IL-17A production and/or in dissipating lymphoid aggregates are predicted in rheumatoid synovia.

The temporal stability of GC^+^ ELS in inflamed synovium remains controversial [39–41]. Indications are that synovial lymphocyte aggregation is a dynamic process [42]. In our study, the availability of paired (and temporally separated) synovia was limited, thus restricting the ability to address this possibility. Separate synovia obtained from a single patient, 5 months apart, were classified into opposing *IL17A*/*CD21L* subgroups. In the paired synovia from this patient and from another two patients that also classified differently over time, gains in expression of *IL17A* and/or *CD21L* were the most notable feature. Whether this represents spontaneous behaviour of synovial aggregates, response (or lack thereof) to pharmacological treatments is unclear. Equally, whether *IL17A*^+^/*CD21L*^+^ and *IL17A*^-^/*CD21L*^-^ synovia co-exist at the same time, within individual patients remains unknown.

Amongst those tested, the majority (~84%) within our patient cohort were ACPA^+^. Synovia from ACPA^+^ or ACPA^-^ patients were not confined to a particular *IL17A*/*CD21L* synovial subgroup. Consequently, we were unable to replicate data that link greater synovial B cell infiltrates and lymphoid aggregates with ACPA positivity [43]. The comparatively smaller sample size, the higher percentage of synovia from ACPA^+^ patients within our cohort and longer disease duration of patients in our study potentially contribute to this anomaly.

While *IL17A*^+^/*CD21L*^+^ or *IL17A*^-^/*CD21L*^-^ synovia are essentially polarised subgroups, the remaining two subgroups (i.e. *IL17A*^+^/*CD2L*^-^ or *IL17A*^-^/*CD21L*^+^) that lack either *IL17A* or *CD21L* expression, display “intermediate” levels of lymphoid aggregation. However, it is unknown if these are transitional stages of synovial inflammation, progressing towards larger aggregates, or whether they represent a regression from these states. Together, the *IL17A*^+^/*CD21L*^-^ and *IL17A*^-^/*CD21L*^+^ subgroups share comparable measures of *IL23A* expression, numbers and size of any lymphoid aggregates. However, by definition these are two molecularly distinct synovial subgroups. More information is required that deciphers the complexity of these synovia beyond expression of the *IL17A* and *CD21L* genes before they can be established as independent inflammatory stages.

Microarray analysis established distinct gene expression profiles for the more polarised *IL17A*^+^/*CD21L*^+^ and *IL17A*^-^/*CD21L*^-^ synovia. These profiles indicate generally heightened immune activity in *IL17A*^+^/*CD21L*^+^ synovia including processes and pathways dominated by B-lymphocytes and their immune-mediated functions. Indeed, the profile for *IL17A*^+^/*CD21L*^+^ synovia is consistent with that previously described for “high inflammatory” synovia [44] and includes select genes (e.g. *CD38*, *IGK*, *XBP1*, *MS4A1*, *CD19*, *SLAMF6*, C*XCL13*) previously associated with a lymphoid synovial pathotype [1]. Amongst these genes there is growing evidence that CXCL13 directs B-lymphocyte accumulation and aggregation within synovium [1, 44, 45]. We also observed increased *RGS13* expression in *IL17A*^+^/*CD21L*^+^ synovia, which is compatible with the mechanisms downstream of CXCL13-ligand and CXCR5-receptor interactions, driving this recruitment process [45, 46]. However, we have not had the opportunity to confirm promising indications [1] that circulating levels of CXCL13 provide a non-invasive indication of B-lymphocyte dominated synovial inflammation. In contrast, gene expression within *IL17A*^-^/*CD21L*^-^ synovia suggests bone and cartilage morphogenesis or remodelling is a feature. The profile here, including genes such as *DKK3*, *TNRRSF11B*/osteoprotegerin, *WNT9B*, and *FGFR1*, resembles that for “low inflammatory” synovia previously described [1, 44].

In summary, measures of *IL17A* and *CD21L* gene expression provide a molecular basis for the classification of rheumatoid synovia that in part accommodates histological differences. The lack of clinical correlation with the molecular data is consistent with published data [41, 42]. and it seems this classification is also unlikely to predict outcome in RA. However, the possibilities that *IL17A*^+^/*CD21L*^+^ synovia are more likely in ACPA^+^ patients, or more easily predicted through measures of circulating CXCL13 require future investigation. Our data indicate that a distinct inflammatory process accompanies *IL17A* and *CD21L* co-expression. Clearly B-cells are involved and their prominence indicates that therapies targeting B-cells, such as rituximab, are likely to be more efficacious towards *IL17A*^+^/*CD21L*^+^ synovia. More detailed knowledge of the synovial inflammation associated with *IL17A*^+^ and *CD21L*^+^ expression should highlight additional targets and might offer the future prospect of selecting biological therapy based on the definition of these different types of joint synovial inflammation.

## References

[1] Dennis G Holweg CTJ, Kummerfeld SK, Choy DF, Setiadi AF, Hackney JA, et al Synovial phenotypes in rheumatoid arthritis correlate with response to biologic therapeutics. Arthritis Res Ther. 2014; 16: R90 10.1186/ar4555 25167216 PMC4060385

[2] Pitzalis C, Kelly S, Humby F. New learnings on the pathophysiology of RA from synovial biopsies. Curr Opin Rheumatol. 2013; 25: 334–344. 10.1097/BOR.0b013e32835fd8eb 23492740

[3] Manzo A, Bombardieri M, Humby F, Pitzalis C. Secondary and ectopic lymphoid tissue responses in Rheumatoid Arthritis: from inflammation to autoimmunity and tissue damage/remodelling. Immunol Rev. 2010; 233: 267–285. 10.1111/j.0105-2896.2009.00861.x 20193005

[4] Krautler NJ, Kana V, Kranich J, Tian Y, Perera D, Lemm D, et al Follicular Dendritic Cells Emerge from Ubiquitous Perivascular Precursors. Cell. 2012; 150: 194–206. 10.1016/j.cell.2012.05.032 22770220 PMC3704230

[5] Noort AR, van Zoest KP, van Baarsen LG, Maracle CX, Helder B, Papazian N, et al Tertiary lymphoid structures in Rheumatoid Arthritis: NF-kB-inducing kinase-positive endothelial cells as central players. Am J Pathol. 2015; 185: 1935–1943. 10.1016/j.ajpath.2015.03.012 25963989

[6] Finch DK, Ettinger R., Karnell JL., Herbst R., Sleeman MA. Effects of CXCL13 inhibition on lymphoid follicles in models of autoimmune disease. Eur J Clin Invest. 2013; 43: 501–509. 10.1111/eci.12063 23517338

[7] Humby F, Bombardieri M, Manzo A, Kelly S, Kirkham B, Spencer J, et al Ectopic lymphoid structures support ongoing production of class-switched autoantibodies in rheumatoid synovium. PLoS Med. 2009; 6: e1. 10.1371/journal.pmed.0060001 PMC2621263 19143467

[8] Liu YJ, de Bouteiller O, Parham CL, Grouard G, Djossou O, de Saint-Vis B, et al Follicular dendritic cells specifically express the long CR2/CD21 isoform. J Exp Med. 1997; 185: 165–170. 8996252 10.1084/jem.185.1.165 PMC2196095

[9] Scheel T, Gursche A, Zacher J, Haupi T, Berek C. V-region analysis of locally defined synovial B and plasma cells reveals selected B cell expansion and accumulation of plasma cell clones in rheumatoid arthritis. Arthritis Rheum. 2011; 63: 63–71. 10.1002/art.27767 20882667

[10] Takemura S, Braun A, Crowson C, Kurtin PJ, Cofiled RH, O’Fallon WM, et al Lymphoid neogenesis in rheumatoid synovitis. J Immunol. 2001; 167: 1072–1080. 11441118 10.4049/jimmunol.167.2.1072

[11] Randen I, Mellbye OJ, Forre O, Natvig JB. The identification of germinal centres and follicular dendritic cell networks in rheumatoid synovial tissue. Scand J Immunol. 1995; 41: 481–486. 7725067 10.1111/j.1365-3083.1995.tb03596.x

[12] Kirkham BW, Lassere MN, Edmonds JP, Juhasz KM, Bird PA, Lee CS, Shnier R, Portek IJ. Synovial membrane cytokine expresion is predictive of joint damage progression in rheumatoid arthritis: a two-year prospective study (the DAMAGE study cohort). Arthritis Rheum. 2006; 54: 1122–1131. 10.1002/art.21749 16572447

[13] Chabaud M, Durand JM, Buchs N, Fossiez F, Page G, Frappart L, et al Human interleukin-17: A T-cell derived proinflammatory cytokine produced by the rheumatoid synovium. Arthritis Rheum. 1999; 42: 963–970. 10.1002/1529-0131(199905)42:5<963::AID-ANR15>3.0.CO;2-E 10323452

[14] Moran EM, Mullan R, McCormick J, Connolly M, Sullivan O, Fitzgerald O, et al Human rheumatoid arthritis tissue production of IL-17A drives matrix and cartilage degradation: synergy with tumour necrosis factor-alpha, Oncostatin M and response to biologic therapies. Arthritis Res Ther. 2009; 11: R113 10.1186/ar2772 19627579 PMC2745795

[15] Koshy PJ, Hendersen N, Logan C, Life PF, Cawston TE, Rowan AD. Interleukin-17 induces cartilage breakdown: novel synergistic effects in combination with proinflammatory cytokines. Ann Rheum Dis. 2002; 61: 704–713. 10.1136/ard.61.8.704 12117676 PMC1754191

[16] Wei M, Duan D. Efficacy and safety of monoclonal antibodies targeting interleukin-17 pathway for inflammatory arthritis: a meta-analysis of randomized controlled clinical trials. Drug Des Devel Ther. 2016; 10: 2771–2777. 10.2147/DDDT.S91374 27672309 PMC5024778

[17] Stamp LK, Easson A, Pettersson L, Higton J, Hessian PA. Monocyte Derived Interleukin (IL)-23 is an Important Determinant of synovial IL-17A expression in Rheumatoid Arthritis. J. Rheumatol. 2009; 36: 2403–2408. 10.3899/jrheum.081304 19797506

[18] Canete JD, Celis R, Yeremenko N, Sanmarti R, van Duivenvoorde L, Ramirez J, et al Ectopic lymphoid neogenesis is strongly associated with activation of the IL-23 pathway in rheumatoid arthritis. Arthritis Res Ther. 2015; 17: 173 10.1186/s13075-015-0688-0 26156866 PMC4496927

[19] Hsu HC, Yang P, Wang J, Wu Q, Myers R, Chen J, et al Interleukin 17-producing T helper cells and interleukin 17 orchestrate autoreactive germinal center development in autoimmune BXD2 mice. Nat Immunol. 2008; 9: 166–175. 10.1038/ni1552 18157131

[20] Kishi Y, Kondo T, Xiao S, Yosef N, Gaublomme J, Wu C, et al Protein C receptor (PROCR) is a negative regulator of Th17 pathogenicity. J Exp Med. 2016; 213: 2489–2501. 10.1084/jem.20151118 27670590 PMC5068226

[21] Nylander AN, Ponath GD, Axisa P-P, Mubarak M, Tomayko M, Kuchroo VK, et al Podoplanin is a negative regulator of Th17 inflammation. J Clin Invest Insight. 2017; 2: e92321 10.1172/jci.insight.92321 28878118 PMC5621890

[22] Gaublomme JT, Yosef N, Lee Y, Gertner RS, Yang LV, Wu C, et al Single cell genomics unveils critical regulators of Th17 cell pathogenicity. Cell. 2015; 163: 1400–1412. 10.1016/j.cell.2015.11.009 26607794 PMC4671824

[23] Wang C, Yosef N, Gaublomme J, Wu C, Lee Y, Clish CB, et al CD5/AIM regulates lipid biosynthesis and restrains Th17 cell pathogenicity. Cell. 2015; 163: 1413–1427. 10.1016/j.cell.2015.10.068 26607793 PMC4671820

[24] Arnett FC, Edworthy SM, Bloch DA, McShane DJ, Fries JF, Cooper NS, et al The American Rheumatism Association 1987 revised criteria for the classification of rheumatoid arthritis. Arthritis Rheum. 1988; 31: 315–324. 3358796 10.1002/art.1780310302

[25] Millier MJ, Stamp LK, Hessian PA. Digital-PCR for gene expression: impact from inherent tissue RNA degradation. Sci Rep. 2017; 7: 943–947. 29222437 10.1038/s41598-017-17619-0 PMC5722939

[26] Yanni G, Whelan A, Felghery C, Bresnihan B. Analysis of cell populations in rheumatoid arthritis synovial tissues. Semin Arthritis Rheum. 1992; 21: 393–399. 1626285 10.1016/0049-0172(92)90040-k

[27] Irizarry RA, Hobbs B, Colin F, Beazer-Barclay YD, Antonellis KJ, Scherf U, et al Exploration, normalization, and summaries of high density oligonucleotide array probe level data. Biostatistics. 2003; 4: 249–264. 10.1093/biostatistics/4.2.249 12925520

[28] Wettenhall JM, Simpson KM, Satterley K, Smyth GK. AffylmGUI: a graphical user interface for linear modelling of single channel microarray data. Bioinformatics. 2006; 22: 897–899. 10.1093/bioinformatics/btl025 16455752

[29] Eisen MB, Spellman PT, Brown PO, Botstein D. Cluster analysis and display of genome-wide expression patterns. Proc Natl Acad Sci. 1998; 95: 14863–14868. 9843981 10.1073/pnas.95.25.14863 PMC24541

[30] Thomas PD, Campbell MJ, Keyariwai A, Mi H, Karlak B, Daverman R. PANTHER: A Library of Protein Families and Subfamilies Indexed by Function. Genome Res. 2003; 13: 2129–2141. 10.1101/gr.772403 12952881 PMC403709

[31] Cho R.J. and Campbell M.J., Transcription, genomes, function. Trends Genet. 2000; 16: 409–415. 10973070 10.1016/s0168-9525(00)02065-5

[32] Jones GW, Hill DG, Jones SA. Understanding imune cells in tertiary lymphoid organ development: It is all starting to come together. Front Immunol. 2016; 7: 401 10.3389/fimmu.2016.00401 27752256 PMC5046062

[33] Van der Heijde DM, van Riel PL, Nuver-Zwart IH, Gribnau FW, vad de Putte LB. Effects of hydroxychloroquine and sulphasalazine on progression of joint damage in rheumatoid arthritis. Lancet. 1989; 1: 1036–1038. 2565997 10.1016/s0140-6736(89)92442-2

[34] Zhang X, Jin J, Peng X, Ramgolam VS, Markovic-Plese S. Simvastin inhibits IL-17 secretion by targeting multiple IL-17-regulatory cytokines and by inhibiting the expression of IL-17 transcription factor RORC in CD4^+^ lymphocytes. J Immunol. 2008; 180: 6988–6996. 18453621 10.4049/jimmunol.180.10.6988

[35] Bombardieri M., Lewis M., Pitzalis C. Ectopic lymphoid neogenesis in rheumatic autoimmune diseases. Nat Rev Rheumatol. 2017; 1: 141–154. 10.1038/nrrheum.2016.217 28202919

[36] Sato K, Suematsu A, Okamoto K, Yamaguchi A, Morishita Y, Kadono Y, et al Th17 funtions as an osteoclastogenic helper T cell subset that links T cell activation and bone destruction. J Exp Med. 2006; 203: 2673–2682. 10.1084/jem.20061775 17088434 PMC2118166

[37] Ding Y., Li J., Wu Q., Yang P., Luo B., Xie S., et al IL-1RA is essential for opitmal localisation of follicular Th cells in the germinal centre light zone to promote autoantibody-producing B cells. J Immunol. 2013; 191: 1614–1624. 10.4049/jimmunol.1300479 23858031 PMC3819396

[38] Yago T, Nanke Y, Kawamoto M, Furuya T, Kobashigawa T, Kamatani N, et al IL-23 induces human osteoclastogenesis via IL-17 in vitro, and anti-IL-23 antibody attenuates collagen-induced arthritis in rats. Arthrits Res Ther. 2007; 9: R996 10.1186/ar2297 17888176 PMC2212562

[39] Manzo A, Paoletti S, Carulli M, Blades MC, Barone F, Yanni G, et al Systematic microanatomical analysis of CXCL13 and CCL21 in situ production and progressive lymphoid organisatin in rheumatoid synovitis. Eur. J. Immunol. 2005; 35: 1347–1359. 10.1002/eji.200425830 15832291

[40] Weyand C, Gorozny J. Ectopic germinal centre formation in rheumatoid synovitis. Ann N J Acad Sci. 2003; 987: 140–149. 10.1111/j.1749-6632.2003.tb06042.x 12727633

[41] Cantaert T, Kolln J, Timmer T, van der Pouw Kraan T, Vandooren B, Thurlings RM, et al Lymphocyte autoimmuity in rheumatoid synnovitis is independent of ectopic lymphoid neogenesis. J Immunol. 2008; 181:785–794. 18566445 10.4049/jimmunol.181.1.785

[42] Van de Sande MG, Thurlings RM, Boumans MJ, Wijbrandts C., Modesti MG, Gerlag DM, et al Presence of lymphocyte aggregates in the synovium of patients with early arthritis in relationship to diagnosis and outcome: is it a constant feature over time? Ann Rheum Dis. 2011; 70: 700–703. 10.1136/ard.2010.139287 21173012

[43] Orr C, Najm A, Biniecka M, McGarry T, Ng C, Young F, et al Synovial phenotype and anti-citullinated peptide antibodies in rheumatoid arthritis patients. Arth Rheum. 2017; 69: 2114–2123. 10.1002/art.40218 28732135

[44] Van Baarsen LGM, Wijbrandts CA, Timmer TCG, van der Pouw Kraan TCT, Tak PP, Verweij CL. Synovial tisue heterogeneity in rheumatoid arthritis in relation to disease activity and biomarkers in peripheral blood. Arth Rheum. 2010; 62: 1602–1607. 20178127 10.1002/art.27415

[45] Armas-Gonzalez E, Dominguez-Luis MJ, Diaz-Martin A, Arce-Franco M, Castro-Hernandez J, Danelon G, et al Rol eof CXCL13 and CCL20 in the recruitment of B cells to inflammatory foci in chronic arthritis. Arthrits Res Ther. 2018; 20 114 10.1186/s13075-018-1611-22 PMC5992813 29880013

[46] Shi GX, Harrison K, Wilson GL, MOratz C, Kehrl JH. RGS13 regulates germinal center B l;ymphocytes responsiveness to CXC chemokine ligand (CXCL)12 and CXCL13. J. Immunol. J. Immunol. 2002; 169: 2507–2515. 12193720 10.4049/jimmunol.169.5.2507

